# Effect of smoking on treatment outcome among tuberculosis patients in Malaysia; a multicenter study

**DOI:** 10.1186/s12889-020-08856-6

**Published:** 2020-06-04

**Authors:** Amer Hayat Khan, Syed Azhar Syed Sulaiman, Mohamed Azmi Hassali, Kashif Ullah Khan, Long Chiau Ming, Omer Mateen, Malik Obaid Ullah

**Affiliations:** 1 Discipline of Clinical Pharmacy, School of Pharmaceutical Sciences, Universiti Sains Malaysia, 11800 Penang, Malaysia; 2 Discipline of Social and Administrative Pharmacy, School of Pharmaceutical Sciences, Universiti Sains Malaysia, 11800 Penang, Malaysia; 3 PAP Rashidah Sa’adatul Bolkiah Institute of Health Sciences, Universiti Brunei Darussalam, Gadong, Brunei Darussalam; 4 Division of Pharmacy, School of Medicine, University of Tasmania, Hobart, TAS Australia; 5 Ministry of Health, Islamabad, Pakistan

**Keywords:** Smoking, Treatment outcome, Tuberculosis

## Abstract

**Background:**

Smoking plays a key role in the development of tuberculosis (TB) infection and is also a predictor of poor TB treatment prognosis and outcomes. The current study was conducted to determine the prevalence of smoking and to assess the effects of smoking on treatment outcomes among TB patients.

**Methods:**

A multi-center retrospective study design was used to collect data from TB patients in four different states of Malaysia, namely Penang, Sabah, Sarawak, and Selangor. The study included medical records of TB patients admitted to the selected hospitals in the period from January 2006 to March 2009. Medical records with incomplete data were not included. Patient demographics and clinical data were collected using a validated data collection form.

**Results:**

Of all patients with TB (9337), the prevalence of smokers was 4313 (46.2%). Among smokers, 3584 (83.1%) were associated with pulmonary TB, while 729 (16.9%) were associated with extrapulmonary TB. Male gender (OR = 1.43, 95% CI 1.30–1.58), Chinese ethnicity (OR = 1.23, 95% CI 1.02–1.49), Sarawak indigenous ethnicity (OR = 0.74, 95% CI 0.58–0.95), urban residents (OR = 1.46, 95% CI 1.33–1.61), employed individuals (OR = 1.21, 95% CI 1.09–1.34), alcoholics (OR = 4.91, 95% CI 4.04–5.96), drug abusers (OR = 7.43, 95% CI 5.70–9.60) and presence of co-morbid condition (OR = 1.27, 95% CI 1.16–1.40) all showed significant association with smoking habits. This study found that 3236 (75.0%) patients were successfully treated in the smokers’ group, while 4004 (79.7%) patients were non-smokers. The proportion of deaths (6.6%, *n* = 283), defaulters (6.6%, *n* = 284) and treatment interruptions (4.7%, *n* = 204) was higher in the smokers’ group.

**Conclusions:**

Smoking has a strong influence on TB and is a major barrier towards treatment success (OR = 0.76, 95% CI 0.69–0.84, *p* < 0.001). Therefore, the findings indicate that smoking cessations are an effective way to decrease treatment failure and drug resistance.

## Background

Global data on tuberculosis (TB) provided by the World Health Organization revealed that 10.0 million people (range, 9.0–11.1 million) were ill with TB in 2018. In addition, in 2018, there were 1.2 million TB fatalities among people who are HIV negative and 251,000 deaths among those who are HIV positive. Since 2007, TB has been the leading cause of death from a single infectious agent, ranking above HIV/AIDS [1]. Smoking and tuberculosis remain two major health concerns worldwide [2]. Some studies from China and India reported that smoking increases the severity and mortality rate of tuberculosis [2, 3]. In 2010, the WHO suggested a stronger focus on preventing exposures to tuberculosis [4]. In addition to cancer and coronary heart disease, numerous studies have identified smoking as a risk factor in the development of TB [5, 6]. Tobacco-associated deaths are projected to increase to 8.4 million by 2020 [7], along with TB, both of which are major sources of mortality and morbidity. Various components of the cigarette smoke such as free radicals of oxygen, acrolein, formaldehyde and carbon monoxide increase the oxidative stress in smokers, thus affecting the bronchial mucosa and increasing the threat of *Mycobacterium tuberculosis* infection [8]. This might be the reason for less effective TB treatment outcomes among specific populations [9, 10]. Although the prevalence of TB in Malaysia decreased significantly compared to the early 1990s, Malaysia was still ranked as an intermediate burden country by the World Health Organization (WHO) in 2018 with an incidence rate of 92/100,000 and estimated mortality rate of 4.9/100,000 population [1].

Despite increasing pieces of evidence showing a strong association between tobacco smoking and TB [1, 11], there are only a few observational studies highlighting the association between smoking and TB treatment outcomes with limited study population [7] and or single-center studies [12, 13] which are difficult to generalize. Previous studies have typically identified potential risk factors for poor results of TB treatment [7, 9, 10]. In this study, the prevalence of smoking in TB patients in Malaysia was determined and the impact that smoking has on the outcomes of tuberculosis treatment was investigated.

## Methods

### Study design and location

A multicenter retrospective study was conducted among the TB patients in four states of Malaysia (Sabah, Sarawak, Selangor, and Penang) — two representative states from west Malaysia and two from peninsular Malaysia — selected based on the highest burden of TB patients [14].

### Inclusion & exclusion criteria

All TB patients (new, relapse, treatment failure and treatment defaulters) of both genders, either with or without co-morbidity, who presented at the chest clinic of Penang hospital from January 2006 to March 2009 were included in the study. Patients with incomplete medical records were removed from the study. Disease classification, treatment protocol and treatment outcomes were defined as per the Malaysian tuberculosis treatment guidelines [15], which are aiming to meet World Health Organization criteria. Sputum smear examinations were done at the end of two, four and 6 months of treatment in new cases and at the end of two, three, five and 8 months in retreatment cases. Sputum examination was repeated again after 1 month if positive at 2 months of treatment. Treatment outcomes initially recorded as cured, treatment completed, defaulted, transferred out, expired and treatment continued were then classified into two categories: successful or unsuccessful treatment. Cured and treatment-completed patients were placed in the successful treatment category, and the remainder was placed in the unsuccessful treatment category. Owing to the main objective of the study, patients were grouped into either ever smokers (those who smoked cigarettes at the time of TB diagnosis or who had previously smoked) or never smokers (those who had never smoked or had smoked less than 100 cigarettes in their lifetime).

### Data collection

A purpose-developed pre-validated data collection form was used to collect demographic and clinical data. Demographic data included patients’ gender, age, weight, ethnicity, residential area, marital status, and smoking and alcohol consumption status. Clinical data collected included clinical presentation, serum biochemistry results, nature of TB case (new, retreatment - failure, default and relapse), site of TB infection (pulmonary or extrapulmonary), presence of the co-morbid condition along with respective medications, results of sputum cultures, and treatment outcomes.

### Statistical analysis

The data were analyzed using the Statistical Package for Social Sciences (SPSS), version 20.0 (SPSS, Inc., Chicago, IL, USA). Percentages and frequencies were used for categorical variables, while means ± standard deviations were calculated for continuous variables. Univariate and multivariate binary regression analyses were used to examine whether tobacco smoking had contributed to poor treatment outcomes in TB patients. The results are presented as *P*-value, odds ratio (OR) and 95% confidence interval (CI). A *P*-value ≤0.05 was considered statistically significant.

## Results

Out of 9337 TB patients, the prevalence of smokers was 4313 (46.2%) while 5024 (53.8%) were non-smokers. The mean age of patients among smokers and non-smokers was 42.18 ± 15.97 and 41.12 ± 17.05 years old, respectively, while the mean weight was 42.02 ± 10.48 and 41.53 ± 9.77 kg, respectively. The male gender was more predominant among both smokers and non-smokers: 3261 (75.6%) and 3181 (63.3%) patients, respectively. There was a high male-to-female ratio in the smoker’s group (3:1). Most of the cases in the smoker group lie in the age group 26–55 years. Malay ethnicity (28.6%, *n* = 1232) followed by Chinese ethnicity (26.7%, *n* = 1153) were in a higher proportion in the smokers’ group. About 2958 (68.6%), 3159 (73.2%) and 3010 (69.8%) smoker patients belong to urban areas, were unmarried and were unemployed, respectively. The prevalence of alcohol and drug abusers was also higher in the smokers’ group than non-smokers’ group: 18.6% (*n* = 802) and 14% (*n* = 602), respectively. The majority of the smoker patients (83.1%, *n* = 3584) had PTB while co-morbidity was observed in 44.2% (*n* = 1907) of patients (Table 1).

**Table 1:** Sociodemographic distribution of TB patients according to smoking habit

Patient Variables	Total ***N*** = 9337	Never smoked ***N*** = 5024	Ever smoked ***N*** = 4313
**Weight (mean ± SD)**	41.75 ± 10.11	41.53 ± 9.77	42.02 ± 10.48
**Gender**			
Male	6442 (69)	3181 (63.3)	3261 (75.6)
Female	2895 (31)	1843 (36.7)	1052 (24.4)
**Age (mean ± SD)**	41.61 ± 16.57	41.12 ± 17.05	42.18 ± 15.97
< 15	265 (2.8)	172 (3.4)	93 (2.2)
16–25	1472 (15.8)	876 (17.4)	596 (13.8)
26–35	1878 (20.1)	1001 (19.9)	877 (20.3)
36–45	1754 (18.8)	872 (17.4)	882 (20.4)
46–55	1674 (17.9)	880 (17.5)	794 (18.4)
56–65	1279 (13.7)	673 (13.4)	606 (14.1)
> 65	1015 (10.9)	550 (10.9)	465 (10.8)
**Race**			
Malay	2504 (26.8)	1272 (25.3)	1232 (28.6)
Chinese	2211 (23.7)	1058 (21.1)	1153 (26.7)
Indian	671 (7.2)	350 (7)	321 (7.4)
Indigenous Sarawak	976 (10.5)	614 (12.2)	362 (8.4)
Indigenous Sabah	1702 (18.2)	1003 (20)	699 (16.2)
Indonesian immigrants	631 (6.8)	372 (7.4)	259 (6)
Philippine immigrants	493 (5.3)	287 (5.7)	206 (4.8)
Others	149 (1.6)	68 (1.4)	81 (1.9)
**Area**			
Urban	5959 (63.8)	3001 (59.7)	2958 (68.6)
Rural	3378 (36.2)	2023 (40.3)	1355 (31.4)
**Marital status**			
Single	6706 (71.8)	3547 (70.6)	3159 (73.2)
Married	2631 (28.2)	1477 (29.4)	1154 (26.8)
**Employment status**			
Employed	2531 (27.1)	1228 (24.4)	1303 (30.2)
Unemployed	6806 (72.9)	3796 (75.6)	3010 (69.8)
**Alcohol drinkers**			
Yes	941 (10.1)	139 (2.8)	802 (18.6)
No	8396 (89.9)	4885 (97.2)	3511 (81.4)
**Intravenous drug user (IVDU)**			
Yes	670 (7.2)	68 (1.4)	602 (14)
No	8667 (92.8)	4956 (98.6)	3711 (86)
**TB-type**			
PTB	7781 (83.3)	4197 (83.5)	3584 (83.1)
EPTB	1556 (16.7)	827 (16.5)	729 (16.9)
**Co-morbidity**			
Yes	3414 (36.6)	1507 (30)	1907 (44.2)
No	5923 (63.4)	3517 (70)	2406 (55.8)

Patient variables were further analyzed based on smoking habits using univariate and multivariate analysis. Multivariate binary logistic regression in Table 2 shows that the odds of smoking showed a significant association with male gender (OR = 1.43, 95% CI 1.30–1.58), Chinese ethnicity (OR = 1.23, 95% CI 1.02–1.49), and Sarawak indigenous ethnicity (OR = 0.74, 95% CI 0.58–0.95). The odds were high and statistically significant with urban residents (OR = 1.46, 95% CI 1.33–1.61), employed individuals (OR = 1.21, 95% CI 1.09–1.34), alcoholics (OR = 4.91, 95% CI 4.04–5.96), drug abusers (OR = 7.43, 95% CI 5.70–9.60) and those with a co-morbid condition (OR = 1.27, 95% CI 1.16–1.40).

**Table 2:** Distribution of TB patients according to smoking habit based on multivariate analysis

Patient variables	Univariate analysis			Multivariate analysis		
	Crude OR	95% CI	***p***-Value	aOR	95% CI	***p***-Value
**Gender**						
Male	1.79	1.64–1.96	**< 0.001**	1.43	1.30–1.58	**< 0.001**
Female	1.00	–		1.00	–	
**Age**						
< 15	0.62	0.48–0.80	**< 0.001**	0.86	0.66–1.14	0.31
16–25	0.75	0.67–0.85	**< 0.001**	0.92	0.81–1.04	0.22
26–35	1.02	0.92–1.13	0.62	–	–	–
36–45	1.22	1.10–1.35	**< 0.001**	1.02	0.91–1.15	0.65
46–55	1.06	0.95–1.18	0.26	–	–	–
56–65	1.05	0.93–1.18	0.35	–	–	–
> 65	0.98	0.86–1.12	0.79	–	–	–
**Race**						
Malay	1.17	1.07–1.29	**< 0.001**	1.10	0.91–1.32	0.31
Chinese	1.36	1.24–1.50	**< 0.001**	1.23	1.02–1.49	**0.02**
Indian	1.07	0.91–1.25	0.37	–	–	–
Indigenous Sarawak	0.65	0.57–0.75	**< 0.001**	0.74	0.58–0.95	**0.02**
Indigenous Sabah	0.77	0.69–0.86	**< 0.001**	1.01	0.83–1.23	0.91
Indonesian immigrants	0.79	0.67–0.94	**0.007**	0.91	0.72–1.16	0.47
Philippine immigrants	0.82	0.68–0.99	**0.04**	0.97	0.75–1.25	0.83
Others	1.39	1.00–1.93	**0.04**	1.30	0.89–1.90	0.16
**Area**						
Urban	1.47	1.35–1.60	**< 0.001**	1.46	1.33–1.61	**< 0.001**
Rural	1.00	–		1.00	–	
**Marital status**						
Single	1.14	1.04–1.24	**0.005**	0.97	0.88–1.07	0.63
Married	1.00	–		1.00	–	
**Employment status**						
Employed	1.33	1.22–1.46	**< 0.001**	1.21	1.09–1.34	**< 0.001**
Unemployed	1.00	–		1.00	–	
**Alcohol drinkers**						
Yes	8.02	6.67–9.66	**< 0.001**	4.91	4.04–5.96	**< 0.001**
No	1.00	–		1.00	–	
**Intravenous drug user (IVDU)**						
Yes	11.82	9.16–15.24	**< 0.001**	7.43	5.70–9.60	**< 0.001**
No	1.00	–		1.00	–	
**TB type**						
PTB	0.96	0.86–1.08	0.56	–	–	
EPTB	1.00	–		–	–	
**Co-morbidity**						
Yes	1.85	1.69–2.01	**< 0.001**	1.27	1.16–1.40	**< 0.001**
No	1.00	–		1.00	–	

Table 3 is based on the clinical signs and symptoms and shows that significant differences exist among both the smokers’ and non-smokers’ groups. Despite this, data analysis indicated that the prevalence of night sweats (1462, 33.9%), coughs (3579, 83%), shortness of breath (1911, 44.3%), fever (2794, 64.8%), weight loss (2969, 68.8%) and loss of appetite (3181, 73.8%) was significantly higher in the smokers’ group.

**Table 3:** Clinical signs & symptoms among smokers and non-smokers patient groups

Symptoms	Total ***N*** = 9337	Never smoked ***N*** = 5024	Ever smoked ***N*** = 4313	***p***-Value
**Night sweats**				
Yes	2962 (31.7)	1500 (29.9)	1462 (33.9)	**< 0.001**
No	6164 (66)	3382 (67.3)	2782 (64.5)	
Unknown	211 (2.3)	142 (2.8)	69 (1.6)	
**Cough**				
Yes	7365 (78.9)	3786 (75.4)	3579 (83)	**< 0.001**
No	1944 (20.8)	1225 (24.4)	719 (16.7)	
Unknown	28 (0.3)	13 (0.3)	15 (0.3)	
**Sputum**				
Yes	5139 (55)	2947 (58.7)	2192 (50.8)	**< 0.001**
No	4035 (43.2)	2000 (39.8)	2035 (47.2)	
Unknown	163 (1.7)	77 (1.5)	86 (2)	
**Shortening of breath**				
Yes	3944 (42.2)	2033 (40.5)	1911 (44.3)	**0.001**
No	5298 (56.7)	2942 (58.6)	2356 (54.6)	
Unknown	95 (1)	49 (1)	46 (1.1)	
**Fever**				
Yes	5940 (63.6)	3146 (62.6)	2794 (64.8)	**0.002**
No	3377 (36.2)	1873 (37.3)	1504 (34.9)	
Unknown	20 (0.2)	5 (0.1)	15 (0.3)	
**Weight loss**				
Yes	6203 (66.4)	3234 (64.4)	2969 (68.8)	**< 0.001**
No	3037 (32.5)	1705 (33.9)	1332 (30.9)	
Unknown	97 (1)	85 (1.7)	12 (0.3)	
**Loss of appetite**				
Yes	6528 (69.9)	3347 (66.6)	3181 (73.8)	**< 0.001**
No	2694 (28.9)	1575 (31.3)	1119 (25.9)	
Unknown	115 (1.2)	102 (2)	13 (0.3)	
**Hemoptysis**				
Yes	2381 (25.5)	1295 (25.8)	1086 (25.2)	**< 0.001**
No	6842 (73.3)	3637 (72.4)	3205 (74.3)	
Unknown	114 (1.2)	92 (1.8)	22 (0.5)	

This study revealed that the overall success rate was 77.5% (*n* = 7240) patients out of which 75% (3236) patients were successfully treated in the smokers’ group while 4004 (79.7%) patients belonged to non-smokers group. The rates of mortality (6.6%, *n* = 283), defaulters (6.6%, *n* = 284) and treatment interruption (4.7%, *n* = 204) were high in the smokers’ group. In multivariate analysis, treatment completion (aOR 0.87, 95% CI 0.78–0.96, *p* < 0.01), death rate (aOR 1.57, 95% CI 1.31–1.89, *p* < 0.001), defaulters (aOR 1.57, 95% CI 1.31–1.89, *p* < 0.001) and treatment interruption (aOR 1.49, 95% CI 1.21–1.84, *p* < 0.001) showed significant associations with smoking habit. The chances of treatment success rate were less for smokers (aOR 0.76, 95% CI 0.69–0.84, *p* < 0.001), as shown in Table 4.

**Table 4:** Unsuccessful treatment outcomes among smokers and non-smokers patients groups with Binary logistic regression

Treatment outcome	Total ***N*** = 9337	Never smoked ***N*** = 5024	Ever smoked ***N*** = 4313	Univariate analysis			Multivariate analysis		
				Crude OR	95% CI	***p***-Value	aOR	95% CI	***p***-Value
**Cured**									
Yes	5319 (57)	2900 (57.5)	2419 (56.1)	0.93	0.86–1.01	0.111	–	–	–
No	4018 (43)	2124 (42.3)	1894 (43.9)	1.00	–		–	–	
**Treatment completed**									
Yes	1924 (20.6)	1117 (22.2)	807 (18.7)	0.80	0.72–0.89	**< 0.001**	0.87	0.78–0.96	**0.01**
No	7413 (79.4)	3907 (77.8)	3506 (81.3)	1.00	–		1.00	–	
**Expired**									
Yes	500 (5.4)	217 (4.3)	283 (6.6)	1.55	1.29–1.86	**< 0.001**	1.57	1.31–1.89	**< 0.001**
No	8837 (94.6)	4807 (95.7)	4030 (93.4)	1.00	–		1.00	–	
**Defaulters**									
Yes	502 (5.4)	218 (4.3)	284 (6.6)	1.55	1.29–1.86	**< 0.001**	1.57	1.31–1.89	**< 0.001**
No	8835 (94.6)	4806 (95.7)	4029 (93.4)	1.00	–		1.00	–	
**Treatment interrupted**									
Yes	369 (4)	165 (3.3)	204 (4.7)	1.46	1.18–1.80	**< 0.001**	1.49	1.21–1.84	**< 0.001**
No	8968 (96)	4859 (96.7)	4109 (95.3)	1.00	–		1.00	–	
**Treatment continued**									
Yes	723 (7.7)	407 (8.1)	316 (7.3)	0.89	0.77–1.04	0.163	–	–	–
No	8614 (92.3)	4617 (91.9)	3997 (92.7)	1.00	–		–	–	
**Overall treatment success**									
Yes	7240 (77.5)	4004 (79.7)	3236 (75)	0.76	0.69–0.84	**< 0.001**	–	–	–
No	2097 (22.5)	1020 (20.3)	1077 (25)	1.00	–		–	–	–

## Discussion

Based on the National Health and Morbidity Surveys in 2006, the prevalence of smoking in Malaysia was 46.5%, as revised in 2015 [12]. The current study reported the same prevalence of smoking, i.e. 46.2%. Previously published studies conducted in Malaysia reported a higher prevalence of smokers than non-smokers among TB patients [11, 13]. The tobacco-tuberculosis association worldwide has been documented with different types of studies [1]. While the exact mechanism is not understood, nicotine may interfere with the host’s immune reaction to *M. tuberculosis* [16].

The present study found that the male gender has a significant association with smoking habits. This could account for the observed differences in TB rates between men and women. A study from China reported that intravenous drug users (IVDUs) were found to be significantly associated with the smokers’ group [17]. An additional study found that excessive intake of alcohol meant a greater chance of *M. tuberculosis* infection [18]. Chronic ethanol intake causes changes in the immune system and an increased risk of bacterial infections [19]. Consistent with the previous findings, the present study found that alcohol use and IVDUs were significantly associated with smoker TB patients.

If smoking is responsible for a decrease in cure rates and an increase in the risk of rapid disease progression and severity then there is a clear immunopathological association between smoking and TB [20, 21]. In the current study, the treatment success rate was low among the smokers’ group. A person who smokes one packet of cigarettes daily inhales 1.12 μg of iron; iron-loading in the alveolar macrophages makes them more susceptible to the growth of *Mycobacterium tuberculosis* [22]. Cigarette smoke increases the threat of *Mycobacterium tuberculosis* infection in multiple ways: declined activity of alveolar macrophage, impairment of mucociliary clearance, decrease in the immune response of pulmonary lymphocytes, modified pulmonary dendritic cells activity, and reduction in the cytotoxic activity of natural killer cells [8]. Smoking also produces an alteration in both natural and acquired cell immunity, affecting macrophages and leukocytes. The effect of oxidative stress is important, as it induces apoptosis in both activated and non-activated macrophages, favoring the multiplication of the bacilli and making the process chronic [23, 24]. This is why tobacco smoking leads to severe clinical illness and eventually death. The current study showed that smokers were more likely to die. A previous study in China reported that the mortality rate was nine times higher among tuberculosis patients who smoked [25] while other studies from South Africa and India concluded that the higher odds of mortality were among smokers [26, 27]. Previous studies had also proved that tobacco smoking as one predictor and risk factors significantly associated with poor adherence and higher default rate in TB care settings [9, 28, 29]. A study from Hong Kong also showed that a smoking habit is a good indicator to evaluate the risk of defaulters from TB treatment under DOTS (OR = 3.00, 95% CI 1.41–6.39, *p* = 0.004) [9]. Patients who default treatment are at greatest risk of developing drug resistance and spreading TB in the community [30]. A recent study from Malaysia recommends that interventions are necessary to improve treatment compliance in TB patients [11]. Improving compliance among smoking TB patients is a great challenge and should be addressed by securing support from families and social organizations as well as providing smoking cessation interventions [11]. Tachfounti et al. studied the association of smoking among TB patients and reported that the TB treatment failure rate was higher among smokers compared to non-smokers (9.1% vs 4.5%, *p* < 0.01) [31]. A few studies tested the effect of smoking on treatment outcomes and concluded that treatment failure was higher among current and ex-smokers, and smoking was significantly associated with low treatment success [2, 32, 33]. Fahrettin Talay et al. evaluated the treatment outcomes among TB patients and identified multiple patient factors that affect TB treatment outcomes [34]. The current study showed that patient mortality is higher among smokers [OR 1.57 (91.31–1.89); *p* < 0.01]. The reason might be due to drug addiction (OR 5.7) and co-morbidities (OR 1.27). The same picture can be drawn from the published articles [33, 35]. Smoking cessation seems to be an essential means of controlling TB epidemics, improving outcomes and drug resistance particularly in high-burden developing countries, as published elsewhere [33–35].

## Conclusion

In conclusion, smoking is a risk factor for the occurrence of tuberculosis. Additionally, this study shows that the treatment effects are also poor. Tobacco also poses a strong risk factor in terms of poor adherence and higher mortality as well as defaulter rate. Therefore, proper public awareness campaigns and smoking cessation interventions should be presented to all smoking TB patients undergoing DOTS treatment to avoid the severity and progression of the disease and to avoid drug resistance.

### Limitation of the study

A number of TB patients were excluded from the analysis due to a lack of / incomplete smoking-related data.

## Data Availability

The datasets generated and/or analyzed during the current study are not publicly available as institutional permission is required, but are available from the corresponding author upon reasonable request.

## Abbreviations

TB: tuberculosis
WHO: World Health Organization
OR: odds ratio
CI: Confidence interval
MREC: Medical Research Ethics Committee
DOTS: Direct Observed Treatment Short course

## References

[1] World Health Organization (2019). Global tuberculosis report.

[2] Gajalakshmi V, Peto R, Kanaka TS, Jha P (2003). Smoking and mortality from tuberculosis and other diseases in India: retrospective study of 43 000 adult male deaths and 35 000 controls. Lancet.

[3] Liu B-Q, Peto R, Chen Z-M, Boreham J, Wu Y-P, Li J-Y, Campbell TC, Chen J-S (1998). Emerging tobacco hazards in China: 1. Retrospective proportional mortality study of one million deaths. BMJ.

[4] Lönnroth K, Jaramillo E, Williams B, Dye C, Raviglione M (2010). Tuberculosis: the role of risk factors and social determinants. Equity, social determinants, and public health programmes.

[5] Hassmiller K (2006). The association between smoking and tuberculosis.

[6] Bates MN, Khalakdina A, Pai M, Chang L, Lessa F, Smith KR (2007). Risk of tuberculosis from exposure to tobacco smoke: a systematic review and meta-analysis. Arch Intern Med.

[7] Ariyothai N, Podhipak A, Akarasewi P, Tornee S, Smithtikarn S, Thongprathum P (2004). Cigarette smoking and its relation to pulmonary tuberculosis in adults. Southeast Asian J Trop Med Public Health.

[8] Underner M, Perriot J (2012). Tabac et tuberculose. Presse Med.

[9] Chang K, Leung C, Tam C (2004). Risk factors for defaulting from anti-tuberculosis treatment under directly observed treatment in Hong Kong. Int J Tuberc Lung Dis.

[10] Thomas A, Gopi P, Santha T, Chandrasekaran V, Subramani R, Selvakumar N, Eusuff S, Sadacharam K, Narayanan P (2005). Predictors of relapse among pulmonary tuberculosis patients treated in a DOTS programme in South India. Int J Tuberc Lung Dis.

[11] Dujaili JA, Sulaiman SAS, Awaisu A, Muttalif AR, Blebil AQ (2011). Outcomes of tuberculosis treatment: a retrospective cohort analysis of smoking versus non-smoking patients in Penang*,* Malaysia. J Public Health.

[12] Lim HK, Ghazali SM, Kee CC, Lim KK, Chan YY, Teh HC, Yusoff AFM, Kaur G, Zain ZM, Mohamad MHN (2013). Epidemiology of smoking among Malaysian adult males: prevalence and associated factors. BMC Public Health.

[13] Awaisu A, Nik Mohamed MH, Abd Aziz N, Syed Sulaiman SA, Mohamad Noordin N, Muttalif AR, Ahmad Mahayiddin A (2010). Tobacco use prevalence, knowledge, and attitudes among newly diagnosed tuberculosis patients in Penang state and Wilayah Persekutuan Kuala Lumpur*,* Malaysia. Tob Induc Dis.

[14] Amer HK, Syed ASS, Madeeha L, Mohamed AH, Abdul RM, Zohra B, Long CM, Bandeh AT (2019). Treatment outcomes and risk factors of extra-pulmonary tuberculosis in patients with co-morbidities. BMC Infect Dis.

[15] Ministry of Health, Malaysia (MoHM) (2012). Clinical practice guidelines; management of tuberculosis.

[16] Kolappan C, Gopi P (2002). Tobacco smoking and pulmonary tuberculosis. Thorax.

[17] Leung CC, Li T, Lam TH, Yew WW, Law WS, Tam CM, Chan WM, Chan CK, Ho KS, Chang KC (2004). Smoking and tuberculosis among the elderly in Hong Kong. Am J Respir Crit Care Med.

[18] Fiske CT, Hamilton CD, Stout JE (2008). Alcohol use and clinical manifestations of tuberculosis. J Infect.

[19] Brecher AS, Riley C, Basista MH (1995). Acetaldehyde-modified lysozyme function: its potential implication in the promotion of infection in alcoholics. Alcohol.

[20] Yach D (2000). Partnering for better lung health: improving tobacco and tuberculosis control [unresolved issues]. Int J Tuberc Lung Dis.

[21] Maurya V, Vijayan V, Shah A (2002). Smoking and tuberculosis: an association overlooked. Int J Tuberc Lung Dis.

[22] Mateos F, Brock JH, Pérez-Arellano JL (1998). Iron metabolism in the lower respiratory tract. Thorax.

[23] Aoshiba K, Tamaoki J, Nagai A (2001). Acute cigarette smoke exposure induces apoptosis of alveolar macrophages. Am J Phys Lung Cell Mol Phys.

[24] Behnia M, Robertson KA, Martin WJ (2000). Lung infections: role of apoptosis in host defense and pathogenesis of disease. CHEST J.

[25] Wen C-P, Chan T-C, Chan H-T, Tsai M-K, Cheng T-Y, Tsai S-P (2010). The reduction of tuberculosis risks by smoking cessation. BMC Infect Dis.

[26] Sitas F, Urban M, Bradshaw D, Kielkowski D, Bah S, Peto R (2004). Tobacco attributable deaths in South Africa. Tob Control.

[27] Gupta PC, Pednekar MS, Parkin D, Sankaranarayanan R (2005). Tobacco associated mortality in Mumbai (Bombay) India. Results of the Bombay cohort study. Int J Epidemiol.

[28] Santha T, Garg R, Frieden T, Chandrasekaran V, Subramani R, Gopi P, Selvakumar N, Ganapathy S, Charles N, Rajamma J (2002). Risk factors associated with default, failure and death among tuberculosis patients treated in a DOTS programme in Tiruvallur District, South India, 2000. Int J Tuberc Lung Dis.

[29] Lavigne M, Rocher I, Steensma C, Brassard P (2006). The impact of smoking on adherence to treatment for latent tuberculosis infection. BMC Public Health.

[30] Shamaei M, Marjani M, Chitsaz E, Kazempour M, Esmaeili M, Farnia P, Tabarsi P, Amiri MV, Mirsaeidi M, Mansouri D (2009). First-line anti-tuberculosis drug resistance patterns and trends at the national TB referral center in Iran—eight years of surveillance. Int J Infect Dis.

[31] Tachfouti N, Nejjari C, Benjelloun M, Berraho M, Elfakir S, El Rhazi K, Slama K (2011). Association between smoking status, other factors and tuberculosis treatment failure in Morocco. Int J Tuberc Lung Dis.

[32] Gianti A, Vianello S, Casinghini C, Roncarolo F, Ramella F, Maccagni M, Tenconi MT. The “Quit and Win” campaign to promote smoking cessation in Italy: results and one year follow-up across three Italian editions (2000–2004). Ital J Public Health. 2012;4(1).

[33] Amer HK, Mohammad I, Ayesha K, Raja AA, Tahir MK (2015). Smoking on treatment outcomes among tuberculosis patients. Am J Med Sci.

[34] Talay F, Kumbetli S, Altin S (2008). Factors associated with treatment success for tuberculosis patients: a single center's experience in Turkey. Jpn J Infect Dis.

[35] Khan AH (2014). Smoking and tuberculosis: a worst scenario?. J Mycobac Dis.

